# Quantitative GC–MS Analysis of Artificially Aged Paints with Variable Pigment and Linseed Oil Ratios

**DOI:** 10.3390/molecules26082218

**Published:** 2021-04-12

**Authors:** Eliise Tammekivi, Signe Vahur, Martin Vilbaste, Ivo Leito

**Affiliations:** Institute of Chemistry, Faculty of Science and Technology, University of Tartu, Ravila 14a, 50411 Tartu, Estonia; signe.vahur@ut.ee (S.V.); martin.vilbaste@ut.ee (M.V.); ivo.leito@ut.ee (I.L.)

**Keywords:** GC–MS, pigment, linseed oil, derivatisation, quantification, P/S ratio, A/P ratio, ∑D

## Abstract

In this study, quantitative gas chromatography–mass spectrometry (GC–MS) analysis was used to evaluate the influence of pigment concentration on the drying of oil paints. Seven sets of artificially aged self-made paints with different pigments (yellow ochre, red ochre, natural cinnabar, zinc white, Prussian blue, chrome oxide green, hematite + kaolinite) and linseed oil mixtures were analysed. In the pigment + linseed oil mixtures, linseed oil concentration varied in the range of 10 to 95 g/100 g. The results demonstrate that the commonly used palmitic acid to stearic acid ratio (P/S) to distinguish between drying oils varied in a vast range (from especially low 0.6 to a common 1.6) even though the paints contained the same linseed oil. Therefore, the P/S ratio is an unreliable parameter, and other criteria should be included for confirmation. The pigment concentration had a substantial effect on the values used to characterise the degree of drying (azelaic acid to palmitic acid ratio (A/P) and the relative content of dicarboxylic acids (∑D)). The absolute quantification showed that almost all oil paint mock-ups were influenced by pigment concentration. Therefore, pigment concentration needs to be considered as another factor when characterising oil-based paint samples based on the lipid profile.

## 1. Introduction

Paints are complex mixtures that may consist of various organic and inorganic compounds (pigments, binders, fillers, and additives). During the drying/curing of the liquid mixture, the binding material goes through different chemical reactions (oxidation, cross-linking, hydrolysis of ester bonds, polymerisation), by which a solid paint layer is formed. Therefore, identifying the binder may be very challenging due to the loss of original compounds and the appearance of new ones [1,2,3].

One type of binding material whose identification still puzzles scientists are drying oils [4,5,6]. For example, the most common drying oils (linseed, poppy, and walnut oil) consist of the same fatty acids (palmitic, stearic, oleic, linoleic, and linolenic acid) bound together in triglyceride molecules. The only difference is the percentages of the before-mentioned fatty acids [7]. It was discovered by Mills in the 1960s that the ratio of the contents of two saturated fatty acids—palmitic acid to stearic acid (P/S) ratio—is approximately stable during the drying process [8]. Since then, the P/S ratio has been used as one of the main criteria to differentiate between drying oils. Over the years, the P/S value for linseed oil has been reported to be around 1.4–2.4, for walnut oil, 2–4.5, and poppy oil, 3–8 [9]. However, some studies have questioned the stability of the P/S ratio [3,10,11]. For example, Schilling et al. [11] demonstrated that palmitic acid is around four times more prone to evaporate than stearic acid from a drying oil-based paint, which could lead to a decrease in the P/S ratio.

In a study by Keune et al. [10], the P/S ratio was monitored in oil paint mock-ups (artificially created paint samples) made with linseed oil mixed with different pigments. They observed that depending on the pigment, the P/S ratio ranged from 0.8 (paints containing vine black or vermilion) to 1.7 (Naples yellow paints). However, this wide range of the observed P/S ratios could have been caused by the experimental conditions applied for the artificial ageing (light and high relative humidity) because, uncommonly, the non-pigmented linseed oil was reliquified during ageing.

Together with the P/S ratio, ratios of other fatty acids or dicarboxylic acids have been used to characterise the binding material or its degree of oxidation. One is the ratio of azelaic acid to palmitic acid (A/P). The higher the value, the more oxidised/dried is the oil because azelaic acid is one of the main dicarboxylic acids produced from the autoxidation of the unsaturated fatty acids present in the fresh oils [10,12]. Similar to the A/P ratio is the sum of the relative content of dicarboxylic acids to other fatty acids (∑D), which again is higher for more oxidised oil [2]. Both values are used to differentiate drying oils from other lipids such as semi-drying (e.g., canola oil) and non-drying oils (e.g., castor oil) or egg (yolk, white, or whole egg) [13,14,15]. In rough terms, A/P > 1, together with ∑D > 40%, suggests that the binder is a drying oil. In contrast, A/P < 0.3 and ∑D < 15% indicate that the binder is an egg. In the case of a drying oil and egg mixture, the values should be in between [13]. However, numerous studies show that different factors such as the type of the pigment, the origin and pre-treatment of the oil, and environmental conditions affect the fatty acid composition, which in turn influence the P/S, A/P, and ∑D values and make the identification challenging [3,16,17,18,19]. Sometimes, even additives with a similar composition to the fatty acids (such as metal stearates) that have been added to paints affect the previously mentioned values [3,20].

However, one aspect that has been studied less is the effect of pigment concentration on the drying of the oil. To our knowledge, previous research has thoroughly addressed the influence of different pigments [21,22,23,24,25,26,27,28] but neglected the effect of the amount of pigment. The impact of pigment concentration on proteinaceous binders [29] has been studied but not on lipids.

In this study, we have addressed the questions of P/S stability and pigment concentration effect by analysing paint mock-ups in which the pigments and their percentages varied over a wide range (the narrowest pigment range was 25–70 g/100 g and the widest 5–90 g/100 g). Seven sets of aged paint samples were analysed, each containing different commercially available natural or synthetic pigment (chrome oxide green, natural cinnabar, yellow or red ochre, Prussian blue, zinc white, or a mixture of hematite and kaolinite) mixed with linseed oil. For the analysis, gas chromatography combined with mass spectrometry (GC–MS) was chosen because of its reputation as a standard method for identifying and characterising fatty acids in oil-based paints. All the mentioned paint mock-ups were derivatised with acid-catalysed methylation, and the sample preparation was modified to enable both absolute and relative quantification. The values of the most common ratios (P/S, A/P, ∑D) were examined, and the absolute quantification of palmitic, stearic, and oleic acid was performed based on ref [30].

To the best of our knowledge, this is the first time the absolute quantification method has been applied to characterise fatty acids in aged oil paints. Additionally, attenuated total reflection Fourier transform infrared (ATR–FT–IR) spectroscopy was used to support the GC–MS findings.

## 2. Materials and Methods

### 2.1. Materials

Clarified linseed oil was a product of Lefranc & Bourgeois, Paris, France. The pigments zinc white, Prussian blue LUX (45202), natural cinnabar (10620), and chrome oxide green (44200) were obtained from Kremer Pigmente GmbH & Co KG, Aichstetten, BW, Germany. Yellow ochre and red ochre are the product of Kreidezeit Naturfarben GmbH, Lamspringe, NI, Germany (purchased from Safran OÜ, Tartu, Estonia). Kaolinite was obtained from Bang & Bonsomer Group Oy, Helsinki, Finland and hematite from Reakhim.

Methanol (purity ≥ 99.9%), hexane (purity ≥ 97.0%), and toluene (purity ≥ 99.9%) were purchased from Honeywell (Charlotte, NC, USA). Concentrated sulfuric acid (purity 98%) was from VWR Chemicals (Radnor, PA, USA), K_2_CO_3_ (purity 99.5%) from Reakhim, glass wool from Supelco (Bellefonte, PA, Unites States), and hexadecane (purity ≥ 99%) from Sigma-Aldrich (St. Louis, MO, USA). For the absolute quantification, a standard mixture of fatty acid methyl esters (FAME) was purchased from Sigma-Aldrich. The concentrations of FAMEs used in this study were the following: methyl palmitate 9.9%, methyl stearate 5.95%, methyl oleate + methyl elaidate (*Z* + *E*) 34.9%, and their purities were in the range of 99.0–100.0%.

### 2.2. Preparation and Ageing of the Pigment and Linseed Oil Mixtures

This investigation is part of a larger research project related to the quantitative analysis of different painting materials (S. Vahur’s grant PUT1521). All these self-made oil paint mixtures have been made during the framework of this project.

Six sets of pigment (yellow ochre, red ochre, natural cinnabar, zinc white, Prussian blue, or chrome oxide green) + linseed oil mixtures and one set of hematite + kaolinite + linseed oil mixture with different mass ratios were prepared on Petri dishes by weighing components and mixing thoroughly. A diverse set of pigments was chosen. They include both synthetic (Prussian blue, zinc white, chrome oxide green) and natural pigments (natural cinnabar, both ochres, hematite), as well as one that is known to produce metal soaps (zinc white). Among them are slow driers (cinnabar, zinc white), medium driers (chrome oxide green, ochres), and a fast drier (Prussian blue). The red ochre and hematite + kaolinite mixtures can both be called red ochres, but these were analysed to see how much additives affect the drying processes. Each set contained 10 to 16 paint mock-ups in which the approximate concentration of oil varied in the range of 10 to 95 g/100 g. The total initial weight for zinc white + linseed oil and yellow ochre + linseed oil mixtures was 3 g, and for the rest of the mock-ups, 1 g. The exact mass ratios of all the paint mock-ups are presented in the Supplementary Materials.

All the oil paint mock-ups were artificially aged eight to ten months. The Prussian blue, red ochre, chrome oxide green, hematite + kaolinite, and natural cinnabar oil paint mock-ups were artificially aged in a specially made chamber with relative humidity (RH) of 35 ± 10% and temperature of 72 ± 5 °C for six months and 62 ± 5 °C for four months. Zinc white + linseed oil and yellow ochre + linseed oil mixtures were aged eight months at 80 ± 2 °C in a drying oven (Heraeus, Thermo Scientific, Waltham, MA, USA).

After ageing, the paints were pulverised with a ball mill (Mini-mill Pulverisette 23, Fritsch) to obtain a homogeneous mixture. The pulverised paint samples were transferred into 4 mL vials and stored at room temperature. After about nine months, these self-made oil paint samples were analysed with GC–MS, and ATR–FT–IR spectra of the paints with the pigment concentration of 50 g/100 g were recorded.

### 2.3. Derivatisation of the Pigment and Linseed Oil Mixtures

GC–MS analysis of aged oil paints requires derivatisation, during which the dried polymeric structure consisting of polar compounds is converted into less polar and more volatile molecules. Depending on the limitations and question at hand, various derivatisation methods may be preferred [1,30,31]. In this work, acid-catalysed methylation (a technique that uses low-cost reagents and is suitable for the analysis of aged paint) was chosen because of the large sample amount and the high number of paint samples used in these experiments. Additionally, a derivatisation method with m-(trifluoromethyl)phenyltrimethylammonium hydroxide (TMTFTH) reagent was tested because of its suitability for the quantitative GC analysis of fresh oils [30]. However, complications occurred with this reagent when the absolute quantification of fatty acids in 10 mg of dried paint samples was attempted. Even when 0.5 mL (compared to the commonly used 15–50 µL [12,30,32]) was used for the derivatisation, the absolute quantities of fatty acids were lower than the values obtained with the acid-catalysed methylation.

One by one, each pigment + linseed oil set was derivatised and analysed with GC–MS in one series. The used derivatisation method was based on the procedure presented in ref [30], with slight modifications. To make the absolute quantification reliable, 10 to 12 mg of paint sample was weighed into a 15 mL glass vial. As some paint samples (especially chrome oxide green + linseed oil mixtures) remained visually heterogeneous even after pulverisation, pieces as different as possible were carefully selected for the derivatisation to enable the best overview of the whole paint. A total of 2 mL of methanol was added to the weighted portion, and the vial was sonicated for 15 min. Then, 0.4 mL of concentrated H_2_SO_4_ was added carefully to the methanolic solution. The vial with the derivatisation mixture was heated for 3 h at 80 °C in an oven (Heraeus). After that, the solution was allowed to cool to room temperature and then extracted with hexane (3 × 2 mL). The obtained hexane solution containing the methylated analytes was pipetted through a glass pipette filled with a layer of K_2_CO_3_ on top of a glass wool layer into another 15 mL glass vial. The combined hexane extracts were evaporated to dryness under a N_2_ evaporator. Then, 2 mL of toluene was added to the residue and weighed on the analytical balance. The solution was stirred vigorously on a VWR vortex mixer to redissolve the analytes. From these stock solutions, dilutions in toluene were made into 1.5 mL Eppendorf^®^ Safe-Lock PCR clean tubes. If the sample contained at least 70 g of oil per 100 g of the mixture, then 0.16 mL of stock solution and 0.4 mL of toluene were weighed; if the oil content was between 40g to 70 g of oil per 100 g, then 0.32 mL of stock solution and 0.24 mL of toluene were weighed; if the oil content was below 40 g/100 g, then 0.64 mL of stock solution was weighed. To all the solutions, 0.1 mL of internal standard solution (0.136 mg·g^−1^ hexadecane in toluene) was added and weighed. The solutions were mixed, and 50 µL of the solution was pipetted into a chromatographic vial with a conical insert (100 µL). To estimate the reproducibility of the applied derivatisation method, one of the most homogeneous samples (yellow ochre + linseed oil, oil concentration 50 g/100 g) was analysed on five analysis days, together with the other paint sets.

### 2.4. Preparation of the Calibration Solutions

For the absolute quantification, an internal standard method presented in ref [30] was used. To construct the calibration curves, eight calibration solutions from the FAME mixture were made in toluene, and the internal standard solution (0.363 mg·g^−1^ hexadecane in toluene) was added. All the calibration solutions were made by weighing. The concentrations of the compounds necessary for this study were as follows: 0.004 to 0.252 mg·g^−1^ for methyl palmitate, 0.002 to 0.151 mg·g^−1^ for methyl stearate, 0.013 to 0.888 mg·g^−1^ for methyl oleate, and around 0.036 mg·g^−1^ for the internal standard. From the calibration solution, 50 µL was pipetted into a chromatographic vial with a conical insert (100 µL). The calibration solutions were measured in the same GC–MS run with the derivatised pigment + linseed oil mixtures in random order.

### 2.5. Instrumentation

For preparing the paint samples, an analytical balance (Sartorius, Göttigen, NI, Germany, resolution 0.1 mg) had been used to weigh all the paint components. For the weighing the aged paint samples, another analytical balance (Precisa, Dietikon, Switzerland, resolution 0.01 mg) was used.

An Agilent (Santa Clara, CA, USA) 5975C inert XL MSD with a triple-axis detector, connected to an Agilent 7890A GC system with an Agilent G4513A autosampler, was used for the GC–MS analysis. The column was a 30 m × 0.25 mm in diameter, 0.25 µm film thickness Agilent DB-225MS capillary column (50% cyanopropylphenyl/50% methylpolysiloxane stationary phase). The injection volume was 0.5 µL. The temperature of the ion source and mass spectrometer were 230 °C and 280 °C, respectively. The inlet temperature was 300 °C, the splitless mode was used, and the split was opened after 2 min. The oven temperature program was as follows: initial temperature 80 °C, isothermal for 2 min, increased at 20 °C min^−1^ to 200 °C, isothermal for 4 min, increased 5 °C min^−1^ to 220 °C, isothermal for 5 min, and finally 10 °C min^−1^ to 230 °C, isothermal for 12 min, with a total run time of 34 min. The solvent delay was 5.6 min, electron ionisation (EI) with 70 eV was used, and helium 6.0 with a flow rate of 1.5 mL min^−1^ was used as the carrier gas. The analysis was performed in two modes—scan mode, where a total ion chromatogram (TIC) was recorded, and selected ion monitoring (SIM) mode. The scan mode was used for the qualitative analysis, and the mass range of 27–400 *m/z* was scanned. For quantitative analysis, SIM mode was used. At the start of the run, the signal corresponding to *m/z* values of 57 and 71 were measured to detect internal standard (hexadecane), and after 9.5 min, the signal of *m/z* values 55, 74, and 81 were measured (corresponding to the most intense fragments of methylated palmitic, stearic, and oleic acid). The measured chromatograms were analysed with Agilent MSD ChemStation and the mass spectra with NIST (National Institute of Standards and Technology) Mass Spectral Library Search 2.0.

For the ATR–FT–IR analysis, a Thermo Scientific (Waltham, MA, USA) Nicolet 6700 FT–IR spectrometer with a Smart Orbit diamond micro-ATR accessory (refractive index is 2.4 and the diameter of the active sample area is 1.5 mm) was used. The FT–IR spectrometer was equipped with a DLaTGS detector, Vectra aluminium interferometer, and CsI beamsplitter. The recorded wavenumber range was 4000–225 cm^−1^, with a resolution of 4 cm^−1^, and the number of scans 128. Constant purging with dry air was used to protect the spectrometer from atmospheric moisture. Thermo Electron’s OMNIC 9 software was used to collect and process the spectra.

## 3. Results and Discussion

**Figure 1:**
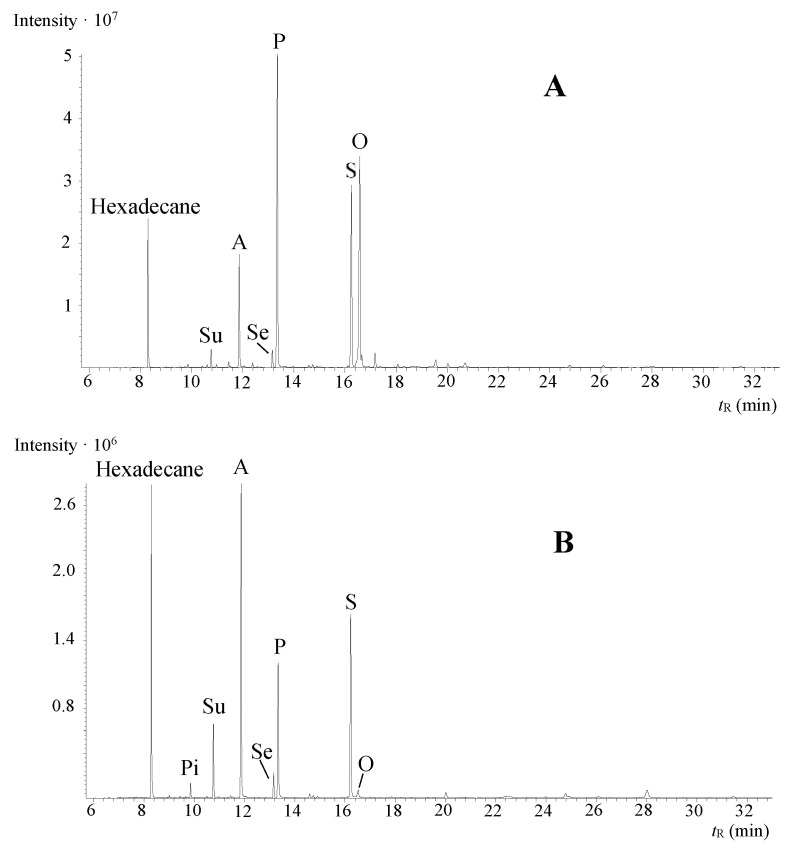
Representative selected ion monitoring (SIM) chromatograms of aged oil paint mock-ups, pigment concentration of 50 g/100 g. (**A**) zinc white and linseed oil and (**B**) Prussian blue and linseed oil. Hexadecane was used as the internal standard, and the abbreviations of other peaks are explained in the text.

The GC–MS analysis of the seven artificially aged paint sets showed that all the samples contained some original fatty acids of linseed oil—palmitic (P), stearic (S), and oleic (O) acid—and the degradation products of the unsaturated fatty acids—azelaic (A), sebacic (Se), suberic (Su), and pimelic acid (Pi). Some of the paint sets contained linoleic and linolenic acid in small quantities (zinc white + linseed oil mixture) and/or other degradation products besides the above-mentioned dicarboxylic acids—9-oxononanoic acid, 10-oxoctadecanoic acid, and undecanedioic acid. Two representative chromatograms − zinc white + linseed oil (A) and Prussian blue + linseed oil (B) − measured in SIM mode are presented in Figure 1.

### 3.1. Relative Quantification

The most common values (P/S, A/P, and ∑D) used to characterise a drying oil were found through relative quantification. The results are presented in Table 1. These values were also found for the same fresh linseed oil that was used to prepare the paint samples: P/S = 1.5, A/P = 0, and ∑D = 0%. The other ratios determined for all paint mock-ups (O/S, A/Su, A/Se) are presented in the Supplementary Materials.

In Table 1, the value of the P/S ratio ranges from 0.6 (Prussian blue + linseed oil) to 1.6 (yellow ochre + linseed oil and zinc white + linseed oil). The P/S ratio had significantly decreased (≤ 1.2) when Prussian blue, chrome oxide green, natural cinnabar, hematite + kaolinite, or red ochre were mixed with the linseed oil. This suggests that with these pigments, palmitic acid is more prone than stearic acid to evaporate during the drying process, which leads to a decrease in the P/S ratio. These results confirm the observations presented in several publications that the P/S ratio may not be the most reliable differentiator [2,3]. Here, we can also conclude that a low P/S ratio does not always imply that additives (e.g., metal stearates) have been added to the paint, as has been sometimes suggested [33]. Further investigation must be conducted to answer whether these pigments could have the same effect on walnut oil (P/S value of fresh oil is 2–4.5) by lowering the P/S value and complicating the differentiation from linseed oil. It is interesting to note that only a few studies see the decrease of the P/S value during paint drying [10,34], and others do not [21,22,26].

**Figure 2:**
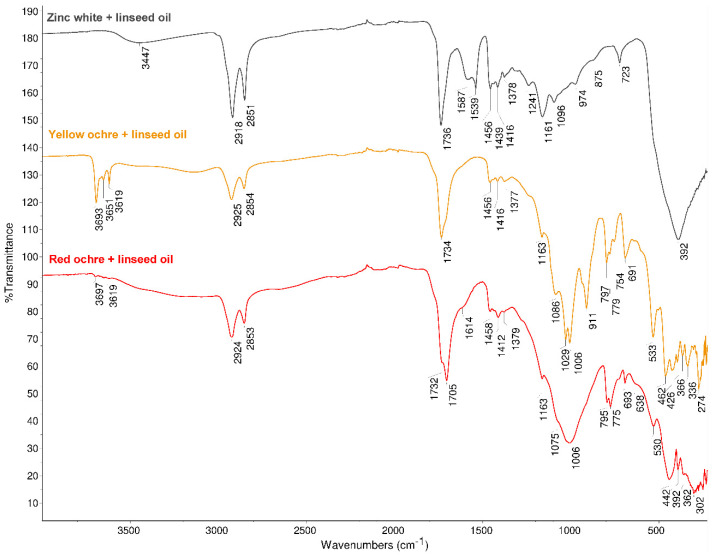
Attenuated total reflection Fourier transform infrared (ATR–FT–IR) spectra of aged paint mock-ups, pigment concentration 50 g/100 g. The spectra of the mixtures containing other pigments (pigment concentration 50 g/100 g) are presented in the Supplementary Materials.

In yellow ochre and zinc white containing oil paint samples, the P/S ratio (average value of 1.6 and 1.5, respectively) is almost the same as the P/S ratio for fresh linseed oil (1.5). The ATR–FT–IR analysis also confirmed that the oil composition of these pigment and linseed oil mixtures differs from the other aged paints. For yellow ochre + linseed oil and zinc white + linseed oil sets, the ATR–FT–IR spectra (Figure 2) differ from the IR spectra of linseed oil mixtures with red ochre (Figure 2), natural cinnabar, Prussian blue, hematite + kaolinite, and chrome oxide green in the wavenumber range of 1530–1750 cm^−1^ (see Figures S1–S4 in the Supplementary Materials). The IR spectra of all the paint samples have an absorption band around 1730–1740 cm^−1^ that belongs to the C=O stretching vibration of the ester group in the triglyceride molecule. However, in the IR spectra of oil paint samples with yellow ochre and zinc white, a C=O stretching band near 1705 cm^−1^ is absent that is present in the spectra of all the other pigment and linseed oil pairs. This absorption has been assigned to carboxylic acids formed during the oxidation of the paint [25]. In the case of zinc white + linseed oil mixture, also absorptions corresponding to zinc carboxylates were observed—1587 cm^−1^ (amorphous structure) and 1539 cm^−1^ (crystalline structure) [35]. The formation of metal carboxylates (or metal soaps) from the metal cation and free carboxylic acids from the hydrolysis of triglycerides is a known phenomenon observed with some pigments, including zinc white [2,24,36]. Importantly, carboxylic acids in the anionic form are not prone to evaporation. Additionally, the absence of 1705 cm^−1^ absorbance in the spectrum of yellow ochre and linseed oil paint shows that also with this pigment, less free carboxylic acids are present. These results imply that the formation of free carboxylic acids (as opposed to carboxylate salts) may lead to the decrease of the P/S value. However, this correlation should be investigated further.

Observing the A/P and ∑D values in Table 1, the highest values are found for red ochre and hematite + kaolinite containing oil paint mock-ups, implying that with these mixtures, the paints are the most oxidised/dried. In most cases, a trend can be observed in the range of one pigment and oil set. The only exception is the hematite + kaolinite + linseed oil paints, in which these values change more hectically over the scope of the set. This could imply that the powdered hematite + kaolinite paint mock-ups were less homogeneous than the other samples.

When observing the results of chrome oxide green, natural cinnabar, yellow ochre, and red ochre containing oil paints, the A/P and ∑D values increase when the linseed oil content decreases. This implies that besides the type of pigment, pigment concentration also influences the drying of the oil. With these pigments, the higher the pigment concentration, the more ‘dried’ is the oil—even though considering one set of pigment and oil mixtures, the samples have been dried over the same period. However, with Prussian blue + linseed oil and zinc white + linseed oil mixtures, these correlations cannot be made, which implies that the pigment concentration does not influence the degree of oil oxidation with these pigments. Therefore, interestingly, the pigment concentration effect is not in correlation with the siccative nature of the pigment. Even though Prussian blue is a known fast drier, the higher concentration of Prussian blue pigment in the paint mixture did not accelerate the drying processes. Likewise, cinnabar is known more as a slow drier; however, the higher concentration of cinnabar pigment accelerated the drying of the paint mock-ups. The fact that with some pigments the A/P and ∑D values increase together with the increase of the pigment concentration, but with other pigments, this correlation is absent leads to another conclusion: the increase in the A/P and ∑D values does indeed come from the higher pigment concentration, not from the fact that the samples contained less oil (one might think that at lower concentration the oil dries better).

As another interesting fact, the A/P and ∑D values are especially low for zinc white + linseed oil mixtures (average values of 0.3 and 13, respectively)—this again may confirm the presence of zinc carboxylates, which hinders the loss of palmitic, stearic, and oleic acid. Therefore, these results show that the A/P ratio does not straightforwardly indicate if the oil has been pre-polymerised (A/P < 0.5 for the zinc white set) or not (A/P > 1 for all the other pigment sets), even though these criteria have been suggested previously [28]. Additionally, here it can be seen that although the A/P ratio is below 0.3 and ∑D is below 15%, the binding material is not an egg. Therefore, in similar cases, those values would lead to incorrect identification of the binding material. These results show that both A/P and ∑D values are affected by the pigment type and, interestingly, in most cases, also by the pigment percentage in the paint mock-up. Therefore, these ratios should only be used cautiously to characterise the state of the dried oil binder.

### 3.2. Absolute Quantification of Fatty Acids

#### 3.2.1. Intermediate Precision Estimation

For estimating the intermediate precision (within-lab reproducibility) of the analysis, one of the yellow ochre and linseed oil mixtures (concentration of oil 50 g/100 g) was analysed on five days, spread over two months (see Table 2). This sample was chosen because the yellow ochre + linseed oil mixtures were one of the most homogeneous, and therefore, the contribution to scatter resulting from sampling should be the lowest. The fatty acids present in the FAME calibration mixture and also in the dried paint samples were methylated palmitic, stearic, and oleic acid, which were quantified with the absolute quantification method.

The results show that acid-catalysed methylation is a suitable derivatisation method with reproducible results for the samples studied in this work. The other aspects of the GC–MS method were the same as in the validated procedure presented by Tammekivi et al. [30]. Therefore, the contents of fatty acids in different pigment and oil mixtures and the parameters derived from them are reliable and can be compared to one another.

#### 3.2.2. Absolute Quantification of the Pigment and Oil Mixtures

Table 3 represents the absolute quantification of palmitic, stearic, and oleic acid in the paint mock-ups. In fresh linseed oil, these values were P = 4.2 g, S = 2.9 g, and O = 15.5 g/100 g. As can be inferred from Table 3, one of the lowest values of these fatty acids were measured for the similar red ochre + linseed oil and hematite + kaolinite + linseed oil mixtures, which agree with the statement suggested above that these paint mock-ups are the most oxidised.

Again, most peculiar are the results of the zinc white-containing oil paint mixtures. In addition to the higher palmitic and stearic acid contents, compared to other pigments throughout the set, the oleic acid content is especially high. This probably contributed to the low A/P and ∑D values because less azelaic acid and other dicarboxylic acids were produced. The contents of palmitic and stearic acid are also the most similar to fresh linseed oil. Interestingly, although natural cinnabar + linseed oil samples (that had 50 g or more linseed oil per 100 g of sample) also had higher oleic acid content than the other paints, the A/P and ∑D values were not remarkably low.

**Figure 3:**
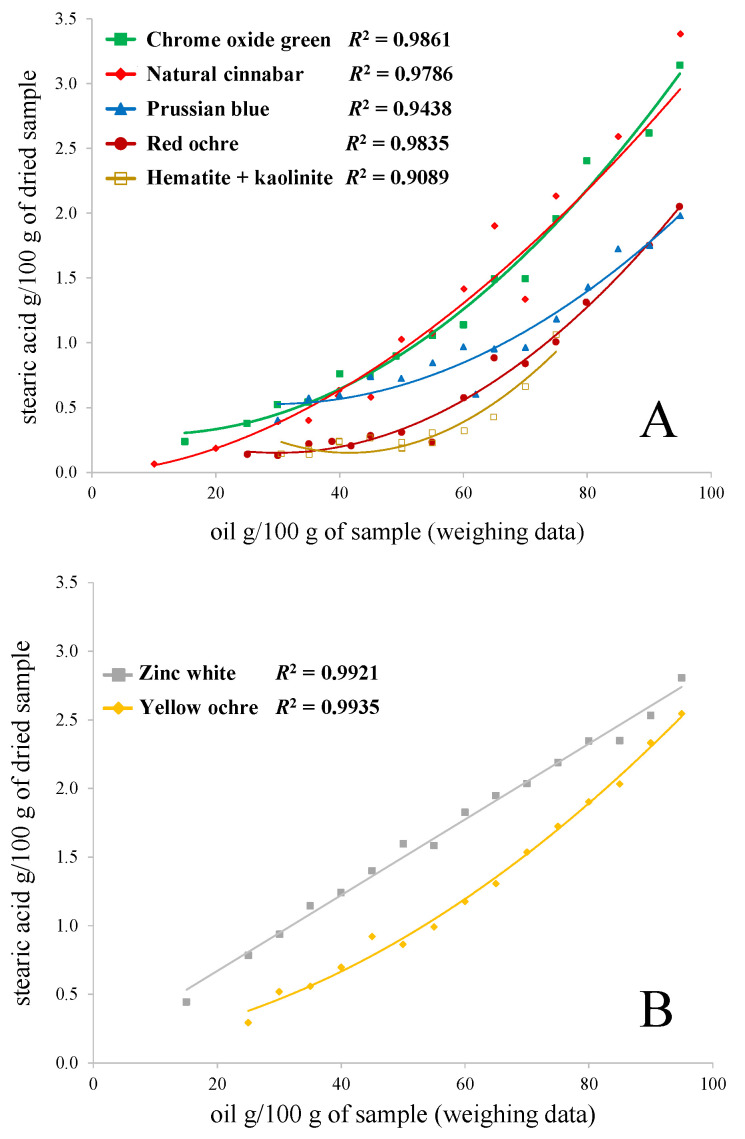
Correlations between stearic acid absolute quantity (g/100 g) vs. oil content (g/100 g) in the weighted sample. (**A**) chrome oxide green, natural cinnabar, Prussian blue, red ochre, and hematite + kaolinite mixtures with linseed oil. (**B**) zinc white and yellow ochre mixtures with linseed oil. The name of the pigment represents the studied pigment and linseed oil mixture.

In Figure 3, the correlations between stearic acid absolute concentration and the pigment concentration in the paint mock-ups are shown. Linseed oil mixtures with chrome oxide green, natural cinnabar, Prussian blue, red ochre, and hematite + kaolinite are presented in Figure 3A; zinc white and yellow ochre oil paint sets are shown in Figure 3B. The same graphs for palmitic acid are presented in the Supplementary Materials. The deviations from the correlation lines in the case of some pigment + linseed oil mixtures (e.g., chrome oxide green and natural cinnabar sets) are likely caused by the inhomogeneities of the samples.

In theory, if the concentration of a pigment does not influence the drying extent of linseed oil, then the correlation should be linear. The correlation in the case of chrome oxide green, natural cinnabar, red ochre, hematite + kaolinite, and Prussian blue are far from linear. This demonstrates that the higher the pigment concentration in these paint samples, the more stearic acid has evaporated from the paint. For zinc white and yellow ochre paints, the curves are the closest to a linear function. In the yellow ochre + linseed oil case, the reason for a curve closer to a linear function could be the fact that the samples were aged two months less, without artificially increased relative humidity, and the amount of the prepared paints was three times higher than for the paint samples presented in Figure 3A. For example, when looking at the absolute values in Table 3 and comparing the stearic acid quantities for 80 g of linseed oil per 100 g of sample to 40 g, it is evident that the decrease has been around 2.7 times, not 2. Therefore, it can be concluded that, also in the case of yellow ochre, the concentration of the pigment influences the drying processes.

Interestingly again, the zinc white + linseed oil paint set acts differently. When comparing the stearic acid quantities of 80 g of linseed oil per 100 g of sample to 40 g, the decrease is two times, which demonstrates that in the case of zinc white paints, the effect of the pigment concentration is the lowest (if present at all). Therefore, in zinc white paint mock-ups, it would be the simplest to suggest the pigment percentage in an unknown sample based on the stearic acid absolute content (if the same value for fresh oil is known).

## 4. Conclusions

In this study, seven artificially aged oil paint mock-ups in varying concentrations were analysed with GC–MS. In addition to the traditional relative quantification of fatty acids, the method developed in our workgroup was successfully used for the absolute quantification of fatty acids in aged paint samples.

The results show that the commonly used ratios to characterise and identify a drying oil (P/S, A/P, and ∑D) vary greatly because of both the different nature of the used pigments and different pigment concentrations. The P/S ratios of Prussian blue, chrome oxide green, natural cinnabar, hematite + kaolinite, and red ochre oil paint sets were generally below 1.0, which is uncommonly low for the used fresh linseed oil with a P/S value of 1.5. On the contrary, the P/S values for yellow ochre and zinc white-containing oil paints remained the same as in fresh oil (1.6 and 1.5, respectively). The high variance in the P/S ratio obtained in this study demonstrates that if this value is to be used to identify/confirm the type of drying oil, it should be performed highly judiciously. The pigment concentration effect could be detected by observing the A/P and ∑D ratios. In the case of chrome oxide green, natural cinnabar, yellow ochre, and red ochre paints, these values increased together with the increase of the pigment concentration. However, for Prussian blue and zinc white, the pigment concentration did not influence the A/P and ∑D values.

The examination of the absolute stearic acid content over the range of paint samples with varying pigment concentration showed that the correlation was far from linear for almost all the pigments (except for zinc white samples). This suggests that higher pigment concentration accelerates the evaporation of stearic acid from the paint samples. Therefore, only the absolute quantification shows that even when the ratios (P/S, A/P, ∑D) are stable or when no trend can be seen, the concentration of the pigment has an influence on the drying processes of oil-paint with almost all the pigments (except zinc white) studied in this work.

## Figures and Tables

**Table 1:** Palmitic acid to stearic acid ratio (P/S), azelaic acid to palmitic acid ratio (A/P), and the sum of the relative content of dicarboxylic acids (∑D) in % of the studied pigment and linseed oil mixtures. The name of the pigment represents the studied pigment and linseed oil mixture.

Oil Concentration in g/100 g (ca. *^a^*)	Chrome Oxide Green			Natural Cinnabar			Red Ochre			Prussian Blue			Hematite + Kaolinite			Yellow Ochre			Zinc White		
	P/S	A/P	∑D	P/S	A/P	∑D	P/S	A/P	∑D	P/S	A/P	∑D	P/S	A/P	∑D	P/S	A/P	∑D	P/S	A/P	∑D
10				1.0	3.0	65.4															
15	0.8	2.3	55.6																1.5	0.4	15.5
20				0.9	2.7	54.5															
25	0.8	2.7	58.4				0.8	6.9	79.8							1.5	1.7	57.5	1.5	0.3	12.4
30	0.7	2.6	57.4	0.8	2.6	52.8	1.0	4.4	72.3	0.7	2.8	58.7	0.9	6.7	74.6	1.4	1.1	46.0	1.5	0.3	12.2
35	0.7	2.8	58.9	0.9	2.3	51.6	0.8	4.8	70.0	0.7	1.7	46.6	1.1	5.6	72.6	1.6	1.2	48.4	1.5	0.3	13.4
39							0.8	5.0	74.4												
40	0.8	2.3	55.6	0.8	2.2	50.4				0.7	2.0	49.3	1.0	3.1	59.0	1.4	1.2	47.0	1.5	0.3	13.1
42							0.9	5.7	75.1												
45	0.8	2.7	59.2	0.8	2.2	50.0	0.8	5.5	73.4	0.7	1.7	46.1	1.0	5.8	73.1	1.4	1.0	41.9	1.5	0.3	13.2
50	0.7	2.7	58.8	0.8	1.5	39.0	0.7	6.8	75.4	0.7	2.1	50.1	0.9	4.2	65.4	1.6	1.0	44.2	1.6	0.3	13.6
55	0.7	2.7	57.2	0.8	1.6	41.9	0.9	6.5	77.1	0.7	2.0	47.0	0.9	3.9	67.1	1.6	0.9	41.1	1.5	0.3	12.6
60	0.8	2.4	56.5	0.9	1.3	36.7	0.8	4.6	69.4	0.7	1.7	44.8	0.9	6.1	73.2	1.6	1.0	42.3	1.5	0.3	12.4
62										0.7	2.6	52.8									
65	0.9	1.7	50.6	0.9	1.2	33.7	0.9	3.1	62.5	0.7	2.7	56.0	1.0	2.0	48.3	1.6	1.0	42.5	1.5	0.3	13.2
70	0.8	2.0	52.5	0.8	1.7	42.4	0.8	3.7	66.4	0.6	2.7	53.8	1.0	4.5	68.8	1.6	0.8	39.7	1.5	0.3	12.3
75	0.9	1.5	46.2	1.0	0.9	28.6	0.9	2.9	62.5	0.7	3.1	58.2	1.0	4.9	70.4	1.6	0.8	37.6	1.5	0.3	12.5
80	1.0	1.2	39.3				0.9	2.5	58.7	0.6	2.9	57.3				1.6	0.7	35.2	1.5	0.3	13.3
85				1.0	0.9	28.4				0.7	2.4	54.1				1.6	0.7	34.4	1.6	0.3	14.4
90	1.0	1.1	36.2				0.9	1.9	49.4	0.7	2.7	57.1				1.6	0.6	30.0	1.5	0.3	14.1
95	1.1	0.8	31.0	1.2	0.5	20.0	0.9	2.0	51.9	0.8	2.4	54.2				1.6	0.6	27.8	1.5	0.3	14.5

*^a^*—These values are rounded. The exact values are presented in the Supplementary Materials.

**Table 2:** Results of absolute quantification of fatty acids in dried yellow ochre and linseed oil mixture (concentration of oil 50 g/100 g) *^a^*.

g/100 g	Palmitic Acid g/100 g	Stearic Acid g/100 g	Oleic Acid g/100 g	P/S
50	1.33 (± 0.05)	0.86 (± 0.01)	0.14 (± 0.03)	0.65 (± 0.02)

*^a^*—The standard deviations are presented in the brackets.

**Table 3:** Absolute quantification of palmitic (P), stearic (S), and oleic (O) acid in g per 100 g of dried oil paint mock-ups. The name of the pigment represents the studied pigment and linseed oil mixture.

Oil Concentration in g/100 g (ca. *^a^*)	Chrome Oxide Green			Natural Cinnabar			Red Ochre			Prussian Blue			Hematite + Kaolinite			Yellow Ochre			Zinc White		
	P	S	O	P	S	O	P	S	O	P	S	O	P	S	O	P	S	O	P	S	O
10				0.1	0.1	0.03															
15	0.2	0.2	0.1																0.6	0.4	0.9
20				0.2	0.2	0.1															
25	0.3	0.4	0.1				0.1	0.1	0.0							0.4	0.3	0.0	1.1	0.8	2.1
30	0.5	0.5	0.1	0.3	0.4	0.2	0.1	0.1	0.0	0.3	0.4	0.0	0.1	0.1	0.1	0.7	0.5	0.02	1.4	0.9	2.5
35	0.5	0.5	0.2	0.5	0.4	0.2	0.2	0.2	0.0	0.4	0.6	0.1	0.1	0.1	0.1	0.9	0.6	0.03	1.6	1.1	3.0
39							0.2	0.2	0.0												
40	0.7	0.8	0.2	0.5	0.6	0.3				0.4	0.6	0.1	0.2	0.2	0.1	1.0	0.7	0.04	1.8	1.2	3.3
42							0.2	0.2	0.1												
45	0.6	0.8	0.2	0.5	0.6	0.3	0.2	0.3	0.1	0.5	0.7	0.1	0.2	0.3	0.1	1.2	0.9	0.1	2.0	1.4	3.8
50	0.6	0.9	0.2	0.9	1.0	0.7	0.2	0.3	0.1	0.5	0.7	0.1	0.2	0.2	0.1	1.3	0.9	0.1	2.3	1.6	4.3
55	0.9	1.1	0.3	0.9	1.1	0.7	0.2	0.2	0.1	0.5	0.8	0.2	0.3	0.3	0.2	1.5	1.0	0.1	2.3	1.6	4.4
60	0.9	1.1	0.3	1.2	1.4	1.4	0.4	0.6	0.2	0.7	1.0	0.2	0.3	0.3	0.2	1.8	1.2	0.2	2.6	1.8	5.1
62										0.4	0.6	0.1									
65	1.3	1.5	0.4	1.6	1.9	1.8	0.7	0.9	0.2	0.6	1.0	0.2	0.4	0.4	0.3	2.0	1.3	0.2	2.8	2.0	5.4
70	1.2	1.5	0.6	1.1	1.3	1.0	0.7	0.8	0.3	0.6	1.0	0.2	0.6	0.7	0.6	2.3	1.5	0.4	2.9	2.0	5.7
75	1.8	2.0	0.8	2.1	2.1	2.3	0.9	1.0	0.3	0.8	1.2	0.3	1.0	1.1	1.0	2.6	1.7	0.5	3.1	2.2	6.1
80	2.3	2.4	1.5				1.1	1.3	0.4	0.9	1.4	0.3				2.8	1.9	0.7	3.4	2.4	6.3
85				2.5	2.6	3.1				1.2	1.7	0.3				3.0	2.0	0.8	3.4	2.4	5.9
90	2.5	2.6	2.3				1.5	1.8	0.9	1.2	1.8	0.3				3.5	2.3	1.5	3.7	2.5	6.3
95	3.6	3.1	3.1	4.0	3.4	5.8	1.8	2.1	0.8	1.5	2.0	0.4				3.8	2.5	2.2	4.1	2.8	6.6

*^a^*—These values are rounded. The exact values are presented in the Supplementary Materials.

## Data Availability

Not applicable.

## Supplementary Materials

The following are available online, **Table S1**: The exact pigment and linseed oil masses in the weighted mixtures *^a^*. **Table S2**: Oleic acid to stearic acid ratio (O/S), azelaic acid to suberic acid ratio (A/Su), and azelaic acid to sebacic acid ratio (A/Se) calculated from the GC–MS analyses. **Figure S1**: ATR–FT–IR spectrum of natural cinnabar and linseed oil aged mixture (50 g/100 g). **Figure S2**: ATR–FT–IR spectrum of Prussian blue and linseed oil aged mixture (50 g/100 g). **Figure S3**: ATR–FT–IR spectrum of hematite + kaolinite and linseed oil aged mixture (25 g of hematite + 25 g of kaolinite per 100 g of paint). **Figure S4**: ATR–FT–IR spectrum of chrome oxide green and linseed oil aged mixture (50 g/100 g). **Figure S5**: Correlations between palmitic acid absolute quantity (g/100 g) vs. oil content (g/100 g) in the weighted sample. (**A**) chrome oxide green, natural cinnabar, Prussian blue, red ochre, and hematite + kaolinite mixtures with linseed oil. (**B**) zinc white and yellow ochre mixtures with linseed oil. The name of the pigment represents the studied pigment and linseed oil mixture.

## Sample Availability

Small amounts of the paint mock-ups are available from Signe Vahur.

## References

[1] Sutherland K. (2018). Gas chromatography/mass spectrometry techniques for the characterisation of organic materials in works of art. Phys. Sci. Rev..

[2] Bonaduce I., Carlyle L., Colombini M.P., Duce C., Ferrari C., Ribechini E., Selleri P., Tinè M.R. (2012). New Insights into the Ageing of Linseed Oil Paint Binder: A Qualitative and Quantitative Analytical Study. PLOS ONE.

[3] Colombini M.P., Modugno F. (2009). Organic Mass Spectrometry in Art and Archaeology.

[4] Castellá F., Pérez-Estebanez M., Mazurek J., Monkes P., Learner T., Niello J.F., Tascón M., Marte F. (2020). A multi-analytical approach for the characterization of modern white paints used for Argentine concrete art paintings during 1940–1960. Talanta.

[5] Andersen C.K., Bonaduce I., Andreotti A., Van Lanschot J., Vila A. (2017). Characterisation of preparation layers in nine Danish Golden Age canvas paintings by SEM–EDX, FTIR and GC–MS. Heritage Sci..

[6] Kasprzok L.M., Fabbri D., Rombolà A.G., Rovetta T., Malagodi M. (2020). Identification of organic materials in historical stringed instruments by off-line analytical pyrolysis solid-phase microextraction with on-fiber silylation and gas chromatography-mass spectrometry. J. Anal. Appl. Pyrolysis.

[7] Winter J., Mills J.S., White R. (1988). The Organic Chemistry of Museum Objects. Stud. Conserv..

[8] Mills J.S. (1966). The Gas Chromatographic Examination of Paint Media. Part I. Fatty Acid Composition and Identification of Dried Oil Films. Stud. Conserv..

[9] Colombini M.P., Andreotti A., Bonaduce I., Modugno F., Ribechini E. (2010). Analytical Strategies for Characterizing Organic Paint Media Using Gas Chromatography/Mass Spectrometry. Accounts Chem. Res..

[10] Keune K., Hoogland F., Boon J.J., Peggie D., Higgit C. (2008). Comparative study of the effect of traditional pigments on artificially aged oil paint systems using complementary analytical techniques. Preprints of 15th Triennal Meeting of ICOM Committee for Conservation.

[11] Schilling M.R., Carson D.M., Khanjian H.P. (1998). Evaporation of Fatty Acids and the Formation of Ghost Images by Framed Oil Paintings, WAAC Newsl. https://cool.culturalheritage.org/waac/wn/wn21/wn21-1/wn21-106.html.

[12] Pitthard V., Stanêk S., Griesser M., Muxeneder T. (2005). Gas Chromatography—Mass Spectrometry of Binding Media from Early 20th Century Paint Samples from Arnold Schönberg’s Palette. Chromatographia.

[13] Kalinina K.B., Bonaduce I., Colombini M.P., Artemieva I.S. (2012). An analytical investigation of the painting technique of Italian Renaissance master Lorenzo Lotto. J. Cult. Heritage.

[14] Izzo F.C. (2010). 20th Century Artists’ Oil Paints: A Chemical-Physical Survey.

[15] Colombini M.P., Modugno F., Giacomelli M., Francesconi S. (1999). Characterisation of proteinaceous binders and drying oils in wall painting samples by gas chromatography–mass spectrometry. J. Chromatogr. A.

[16] Berg J.V.D., Berg K.V.D., Boon J. (2001). Determination of the degree of hydrolysis of oil paint samples using a two-step derivatisation method and on-column GC/MS. Prog. Org. Coatings.

[17] Berg J.D.V.D., Vermist N.D., Carlyle L., Holčapek M., Boon J.J. (2004). Effects of traditional processing methods of linseed oil on the composition of its triacylglycerols. J. Sep. Sci..

[18] Bonaduce I., Ribechini E., Modugno F., Colombini M.P. (2016). Analytical Approaches Based on Gas Chromatography Mass Spectrometry (GC/MS) to Study Organic Materials in Artworks and Archaeological Objects. Top. Curr. Chem..

[19] Colombini M.P., Modugno F., Fuoco R., Tognazzi A. (2002). A GC-MS study on the deterioration of lipidic paint binders. Microchem. J..

[20] La Nasa J., Modugno F., Aloisi M., Lluveras-Tenorio A., Bonaduce I. (2018). Development of a GC/MS method for the qualitative and quantitative analysis of mixtures of free fatty acids and metal soaps in paint samples. Anal. Chim. Acta.

[21] Llorent-Martínez E., Domínguez-Vidal A., Rubio-Domene R., Pascual-Reguera M., Ruiz-Medina A., Ayora-Cañada M. (2014). Identification of lipidic binding media in plasterwork decorations from the Alhambra using GC–MS and chemometrics: Influence of pigments and aging. Microchem. J..

[22] Šefců R., Pitthard V., Chlumská Š., Turková I. (2017). A multianalytical study of oil binding media and pigments on Bohemian Panel Paintings from the first half of the 14th century. J. Cult. Heritage.

[23] Chiavari G., Fabbri D., Prati S. (2005). Effect of pigments on the analysis of fatty acids in siccative oils by pyrolysis methylation and silylation. J. Anal. Appl. Pyrolysis.

[24] Fuster-López L., Izzo F.C., Piovesan M., Yusá-Marco D.J., Sperni L., Zendri E. (2016). Study of the chemical composition and the mechanical behaviour of 20th century commercial artists’ oil paints containing manganese-based pigments. Microchem. J..

[25] Van Der Weerd J., Van Loon A., Boon J.J. (2005). FTIR Studies of the Effects of Pigments on the Aging of Oil. Stud. Conserv..

[26] Gimeno-Adelantado J., Mateo-Castro R., Doménech-Carbó M., Bosch-Reig F., Doménech-Carbó A., Casas-Catalán M., Osete-Cortina L. (2001). Identification of lipid binders in paintings by gas chromatography. J. Chromatogr. A.

[27] Ioakimoglou E., Boyatzis S., Argitis P., Fostiridou A., Papapanagiotou K., Yannovits N. (1999). Thin-Film Study on the Oxidation of Linseed Oil in the Presence of Selected Copper Pigments. Chem. Mater..

[28] Pitthard V., Griesser M., Stanek S. (2006). Methodology and application of gc-ms to study altered organic binding media from objects of the Kunsthistorisches Museum, Vienna. Ann. Chim..

[29] Gautier G., Colombini M.P. (2007). GC–MS identification of proteins in wall painting samples: A fast clean-up procedure to remove copper-based pigment interferences. Talanta.

[30] Tammekivi E., Vahur S., Kekišev O., Van Der Werf I.D., Toom L., Herodes K., Leito I. (2019). Comparison of derivatization methods for the quantitative gas chromatographic analysis of oils. Anal. Methods.

[31] Doménech-Carbó M.T. (2008). Novel analytical methods for characterising binding media and protective coatings in artworks. Anal. Chim. Acta.

[32] Manzano E., Rodríguez-Simón L., Navas N., Checa-Moreno R., Romero-Gámez M., Capitán-Vallvey L. (2011). Study of the GC–MS determination of the palmitic–stearic acid ratio for the characterisation of drying oil in painting: La Encarnación by Alonso Cano as a case study. Talanta.

[33] Banti D., La Nasa J., Tenorio A.L., Modugno F., Berg K.J.V.D., Lee J., Ormsby B., Burnstock A., Bonaduce I. (2018). A molecular study of modern oil paintings: Investigating the role of dicarboxylic acids in the water sensitivity of modern oil paints. RSC Adv..

[34] Modugno F., Di Gianvincenzo F., Degano I., Van Der Werf I.D., Bonaduce I., Berg K.J.V.D. (2019). On the influence of relative humidity on the oxidation and hydrolysis of fresh and aged oil paints. Sci. Rep..

[35] Hermans J.J., Keune K., Van Loon A., Iedema P.D. (2015). An infrared spectroscopic study of the nature of zinc carboxylates in oil paintings. J. Anal. At. Spectrom..

[36] Osmond G. (2012). Zinc white: A review of zinc oxide pigment properties and implications for stability in oil-based paintings. AICCM Bull..

