# Supplementary material for: Quantitative GC–MS Analysis of Artificially Aged Paints with Variable Pigment and Linseed Oil Ratios
Source: Molecules. 2021 Apr 12;26(8):2218. doi: 10.3390/molecules26082218 (PMC8069651; doi:10.3390/molecules26082218)
Supplement: Supplementary file 1 [file molecules-26-02218-s001.pdf]

Supplementary Materials

Quantitative GC-MS Analysis of Artificially Aged Paints with Variable Pigment and Linseed Oil Ratios

Eliise Tammekivi, Signe Vahur, Martin Vilbaste, and Ivo Leito

University of Tartu, Faculty of Science and Technology, Institute of Chemistry, Ravila 14a, 50411 Tartu, Estonia

Table S1. The exact pigment and linseed oil masses in the weighted mixtures [a].

Chrome oxide green	Natural Cinnabar	Yellow ochre	Red ochre	Prussian blue	Zinc white	Hematite + kaolinite		
**oil**	**oil**	oil	oil	oil	oil	oil	hematite	kaolinite
15.00	10.06	25.00	25.09	29.99	15.01	30.58	64.31	5.10
25.02	20.00	30.00	30.02	34.99	25.02	35.09	49.85	15.07
29.96	30.07	35.00	35.03	39.97	30.08	39.92	20.06	40.02
34.87	35.01	40.00	38.75	44.99	35.02	44.91	10.09	44.99
40.02	39.88	45.00	41.85	49.95	39.98	50.06	39.81	10.13
44.91	45.03	49.99	44.99	55.01	44.99	54.96	37.95	7.10
49.09	49.97	55.00	50.00	59.97	50.05	60.10	19.96	19.93
55.00	54.96	60.00	54.94	61.99	55.01	64.88	15.08	20.04
60.00	60.05	65.00	60.05	64.94	59.99	69.99	10.04	19.97
65.00	65.04	70.00	64.95	69.99	65.00	74.98	15.04	9.98
69.98	69.95	74.99	69.99	75.00	70.00			
74.91	74.95	79.98	74.92	80.09	75.02			
79.93	85.01	84.99	79.83	84.98	80.00			
89.96	95.01	89.98	90.01	90.07	85.01			
94.96		94.99	94.89	94.98	90.00			
					95.00			

[a] - In case of chrome oxide green, natural cinnabar, yellow ochre, red ochre, Prussian blue, and zinc white, the mass of linseed oil in g/100 g of paint mixture is presented. The mass of the pigment in g/100 g of mixture can be calculated by subtracting the value presented in the table from 100. For the hematite + kaolinite set the masses of linseed oil, hematite, and kaolinite are presented in g/100 g of paint mixture.

Table S2. Oleic acid to stearic acid ratio (O/S), azelaic acid to suberic acid ratio (A/Su), and azelaic acid to sebacic acid ratio (A/Se) calculated from the GC–MS analyses [a].

oil concentration in g/100 g (ca.[b])	Chrome oxide green			Natural Cinnabar			Red ochre			Prussian blue			Hematite + kaolinite			Yellow ochre			Zinc white		
	O/S	A/Su	A/Se	O/S	A/Su	A/Se	O/S	A/Su	A/Se	O/S	A/Su	A/Se	O/S	A/Su	A/Se	O/S	A/Su	A/Se	O/S	A/Su	A/Se
**10**				0.0	6.4	8.4															
**15**	0.1	5.4	11.3																1.1	10.6	7.2
**20**				0.2	6.8	8.8															
**25**	0.1	6.3	10.7				0.0	5.0	13.2							0.0	4.7	11.7	1.4	9.1	6.1
**30**	0.1	5.7	10.7	0.2	6.3	9.4	0.0	6.3	10.3	0.0	5.2	15.4	0.2	5.2	13.0	0.0	4.4	11.4	1.5	9.0	6.1
**35**	0.1	6.0	10.6	0.3	5.9	9.9	0.1	5.7	11.3	0.1	4.6	12.0	0.3	5.0	10.2	0.0	4.3	12.1	1.4	8.6	6.5
**39**							0.0	6.1	10.8												
**40**	0.1	5.0	10.9	0.2	5.4	9.9				0.1	4.9	12.2	0.5	5.8	10.5	0.1	4.2	12.0	1.5	7.9	6.3
**42**							0.2	5.7	11.0												
**45**	0.1	5.2	10.7	0.2	5.9	9.3	0.1	5.6	11.1	0.1	4.9	12.4	0.3	5.5	10.9	0.1	4.3	11.6	1.5	7.4	6.5
**50**	0.1	5.2	10.5	0.4	6.9	9.6	0.2	5.5	11.3	0.1	4.9	11.7	0.3	6.0	10.5	0.1	4.6	11.2	1.5	7.4	6.1

**55**	0.1	5.2	10.6	0.3	6.1	9.4	0.2	5.8	10.6	0.1	6.4	10.6	0.3	5.6	10.8	0.1	4.7	11.2	1.5	7.6	6.1
**60**	0.1	5.3	10.3	0.5	6.9	9.6	0.1	5.3	11.1	0.1	6.1	10.3	0.2	4.8	10.1	0.1	4.6	11.7	1.5	7.5	6.0
**62**										0.1	6.8	10.0									
**65**	0.1	5.0	10.3	0.5	7.0	9.8	0.1	5.2	11.2	0.1	4.6	11.7	0.5	5.6	10.5	0.1	4.6	11.8	1.5	7.1	6.6
**70**	0.2	5.2	10.1	0.4	5.8	9.6	0.2	5.2	11.0	0.1	6.0	10.9	0.3	5.3	11.0	0.1	4.4	11.3	1.5	7.5	6.3
**75**	0.2	5.0	10.0	0.6	6.9	9.7	0.1	5.2	11.0	0.1	4.6	12.6	0.2	5.9	9.9	0.2	4.9	11.0	1.5	7.0	6.4
**80**	0.3	5.4	9.7				0.1	5.0	10.9	0.1	4.5	12.3				0.2	4.6	11.3	1.5	6.9	6.5
**85**				0.6	6.7	10.1				0.1	4.8	12.1				0.2	4.9	11.2	1.4	6.4	6.9
**90**	0.5	5.6	10.0				0.3	5.4	10.7	0.1	4.6	12.2				0.3	5.2	10.7	1.4	6.6	6.8
**95**	0.5	6.0	9.7	0.9	6.5	10.4	0.2	5.1	10.7	0.1	4.7	11.9				0.4	4.8	10.3	1.3	6.3	7.0

[a] – The name of the pigment represents the studied pigment and linseed oil mixture. [b] – These values are rounded. The exact values are presented in Table S1.

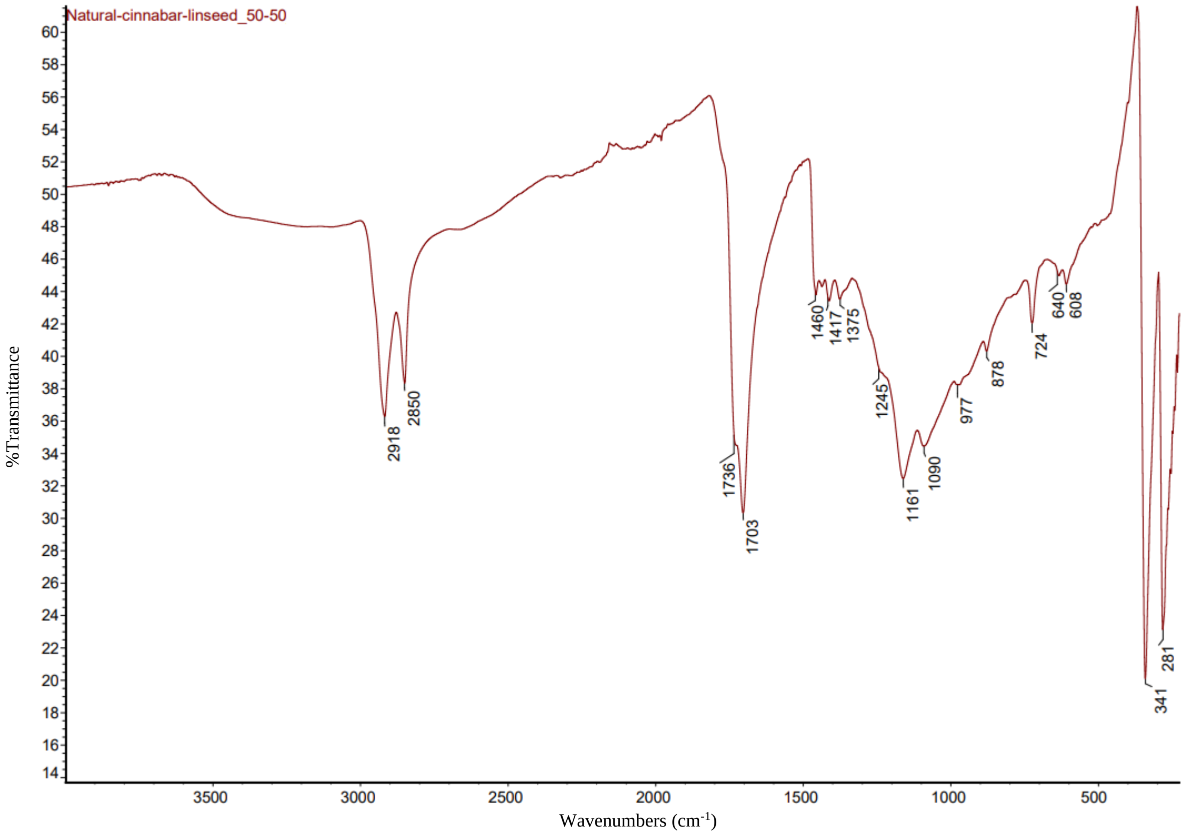

Figure S1. ATR–FT–IR spectrum of natural cinnabar and linseed oil aged mixture (50 g/100 g).

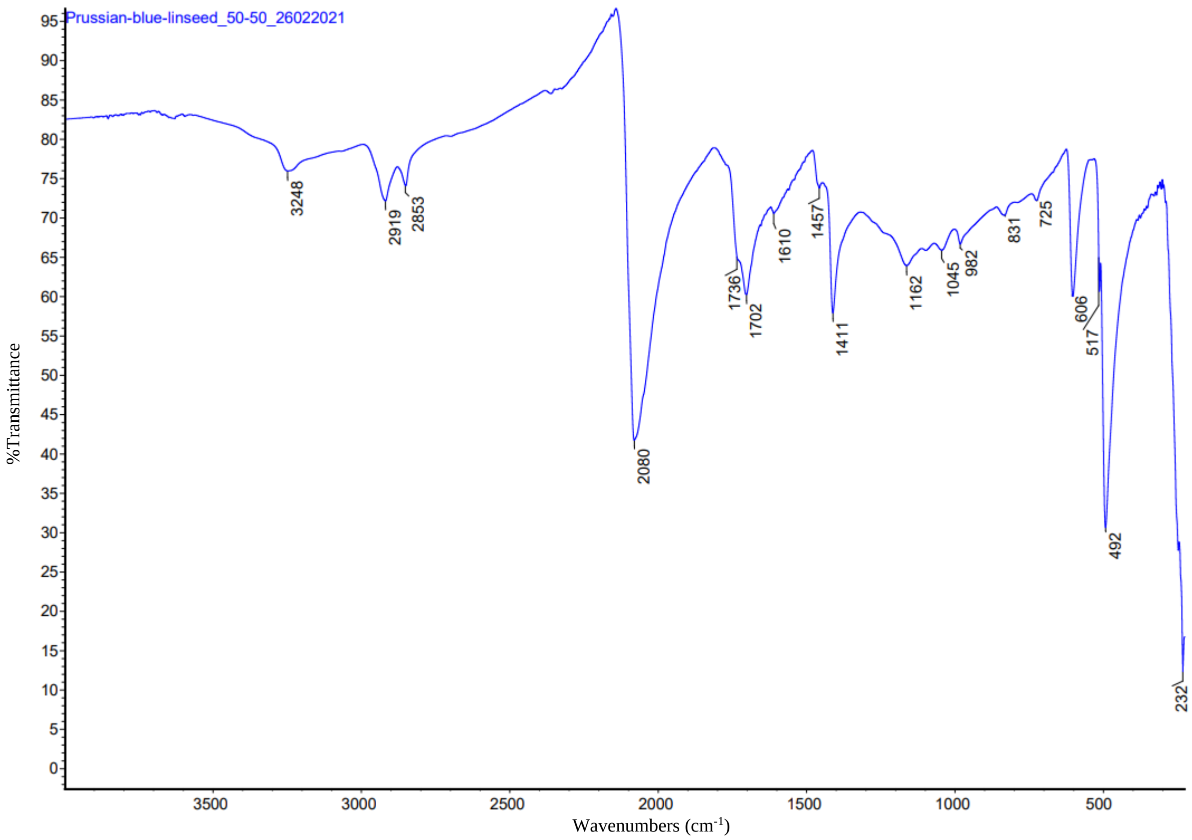

Figure S2. ATR–FT–IR spectrum of Prussian blue and linseed oil aged mixture (50 g/100 g).

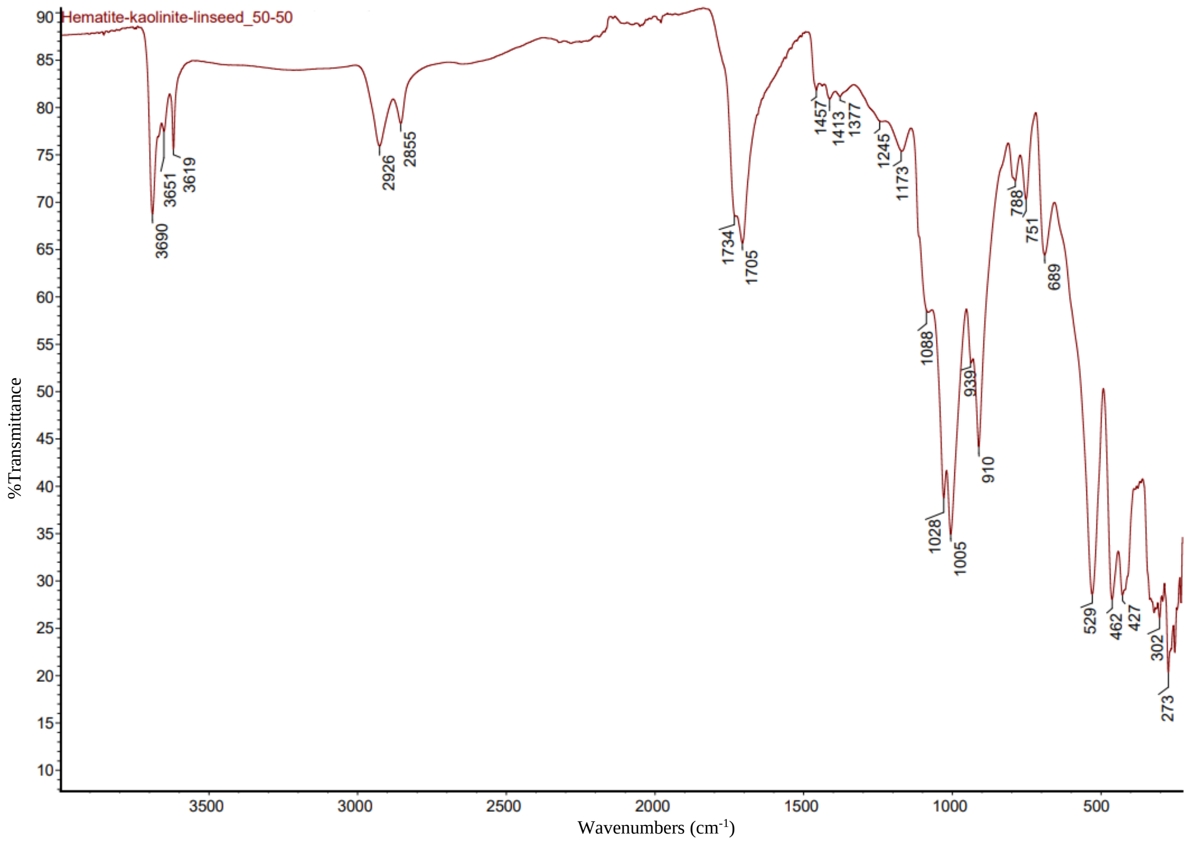

Figure S3. ATR–FT–IR spectrum of hematite + kaolinite and linseed oil aged mixture (25 g of hematite + 25 g of kaolinite per 100 g of paint).

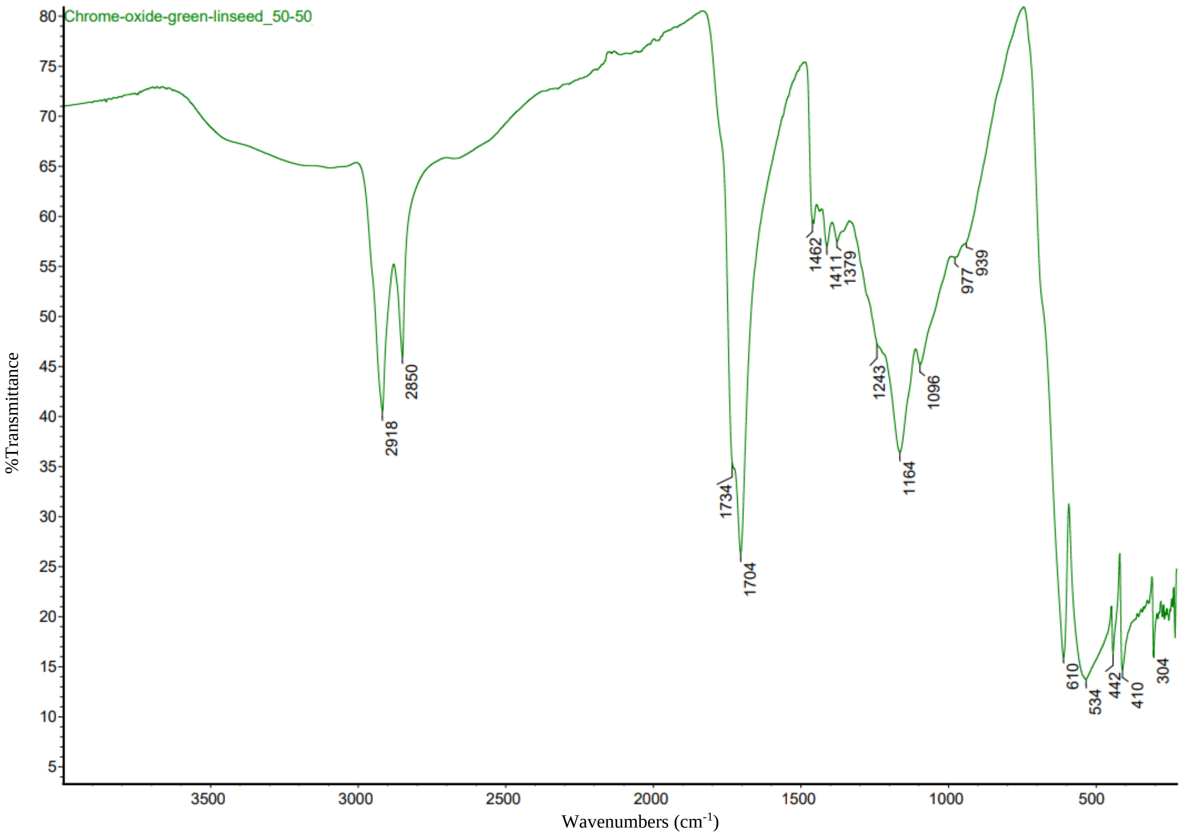

Figure S4. ATR–FT–IR spectrum of chrome oxide green and linseed oil aged mixture (50 g/100 g).

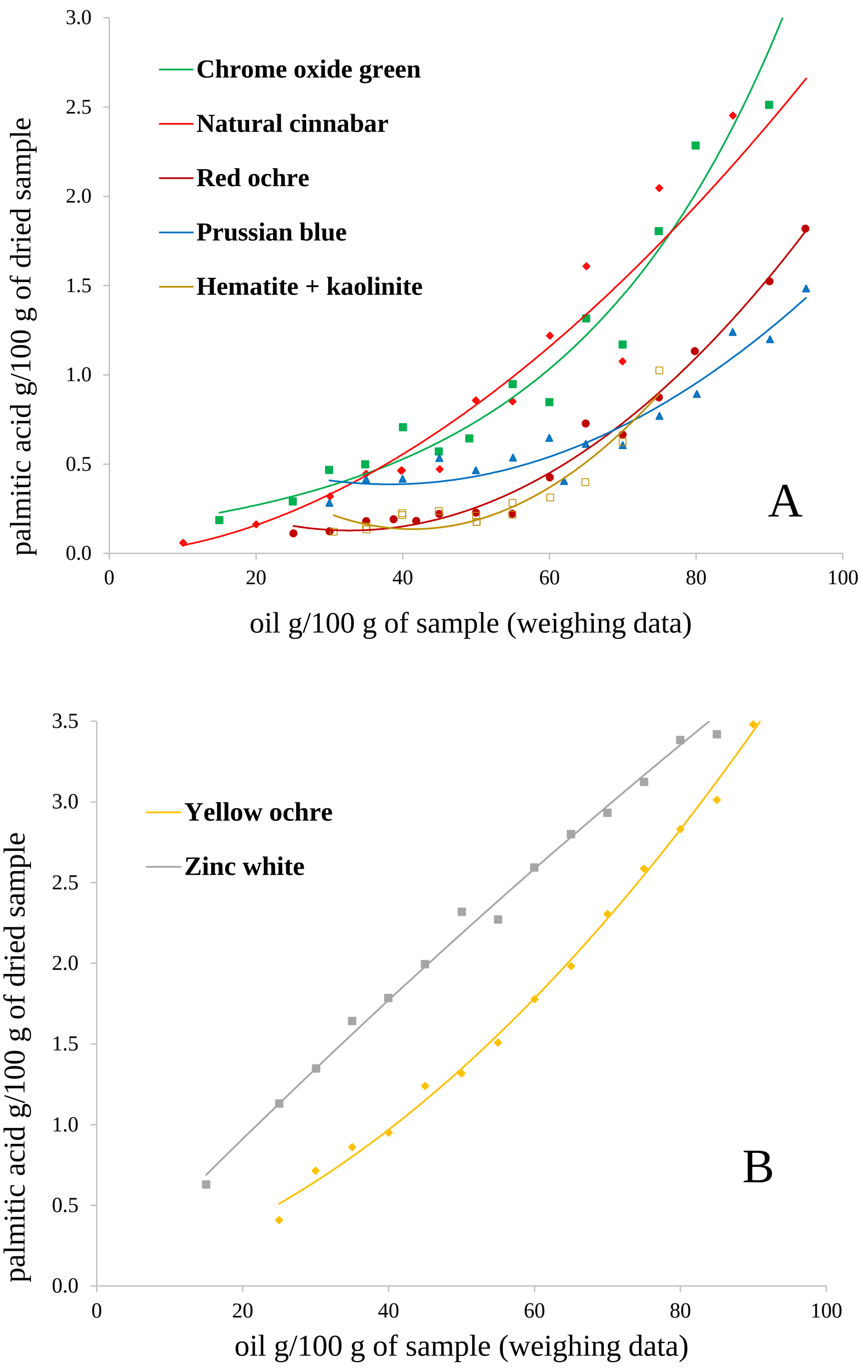

Figure S5. Correlations between palmitic acid absolute quantity (g/100 g) vs. oil content (g/100 g) in the weighted sample. (**A**) chrome oxide green, natural cinnabar, Prussian blue, red ochre, and hematite + kaolinite mixtures with linseed oil. (**B**) zinc white and yellow ochre mixtures with linseed oil. The name of the pigment represents the studied pigment and linseed oil mixture.
